# Supplementary material for: DMRforPairs: identifying Differentially Methylated Regions between unique samples using array based methylation profiles
Source: BMC Bioinformatics. 2014 May 15;15:141. doi: 10.1186/1471-2105-15-141 (PMC4046028; doi:10.1186/1471-2105-15-141)
Supplement: Additional file 2 — DMRforPairs output for the comparison of A431-MCF7 and NA17018-NA17105. Please start from the HTML files in each folder. Available via the BMC Bioinformatics website. [file 1471-2105-15-141-S2.zip › 1394847754114233_MOESM2_ESM/A431_MCF7/figures/32646.pdf]

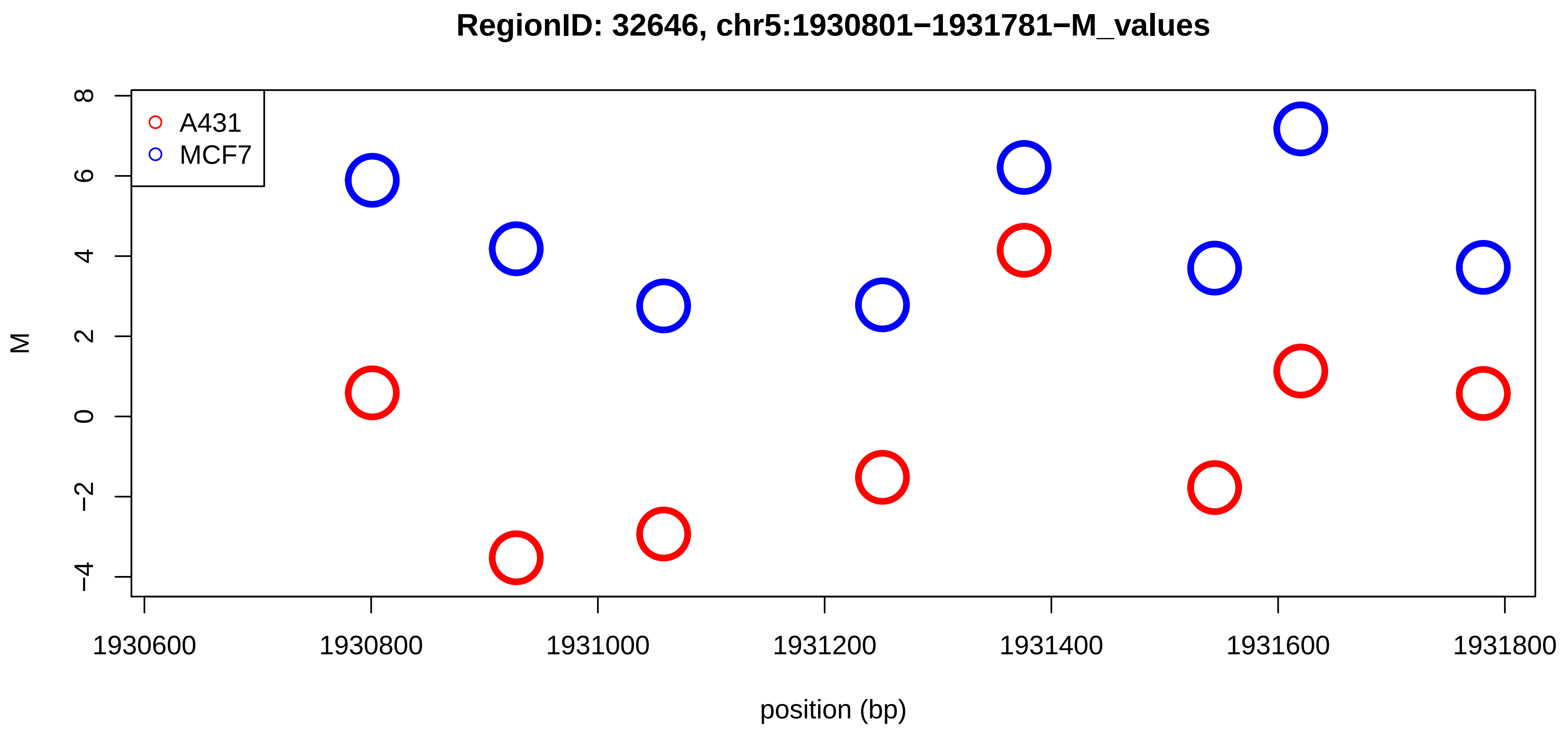
RegionID: 32646, chr5:1930801−1931781−M_values
A431
MCF7
M
−4
−2
0
2
4
6
8
1930600
1930800
1931000
1931200
1931400
1931600
1931800
position (bp)

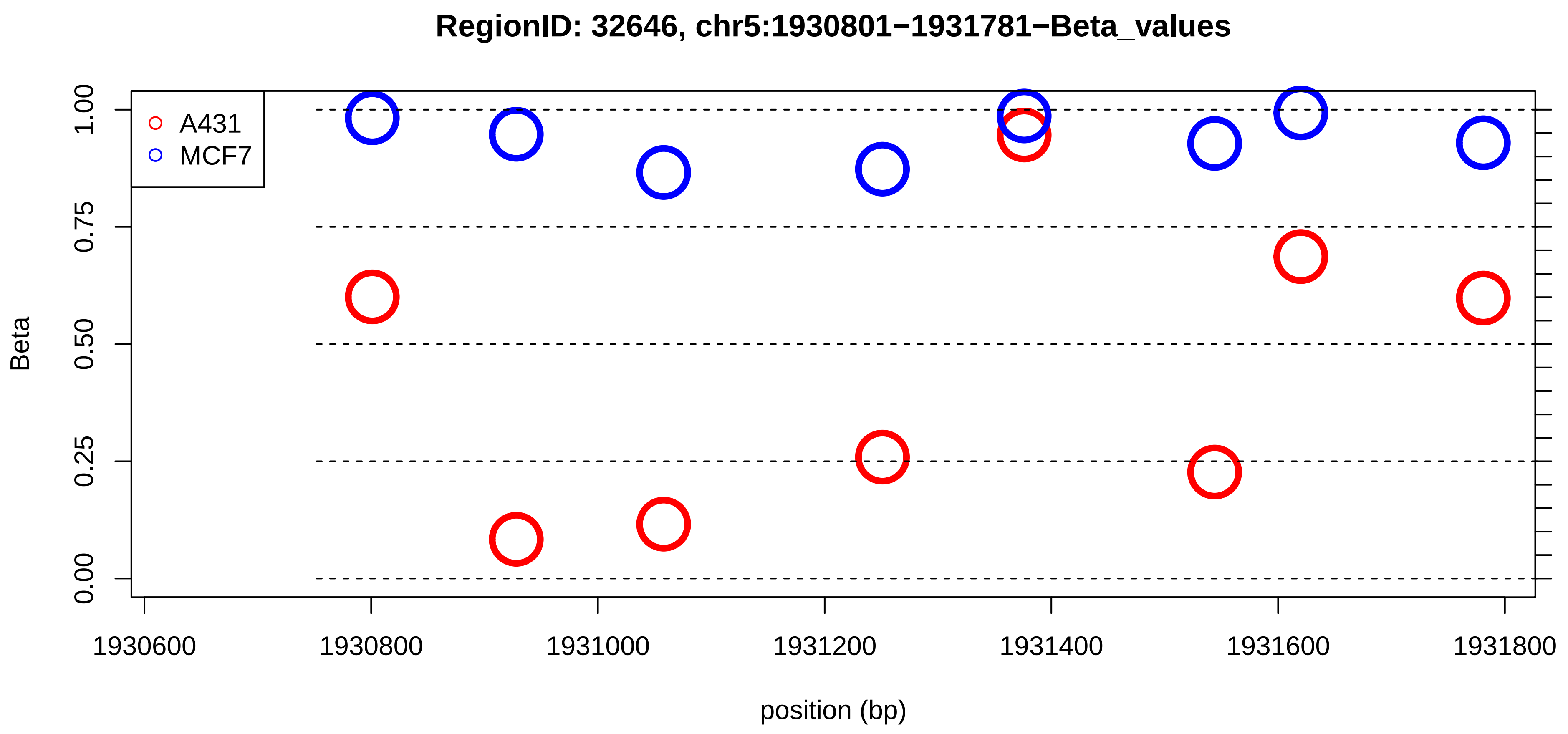
RegionID: 32646, chr5:1930801−1931781−Beta_values
A431
MCF7
Beta
0.00
0.25
0.50
0.75
1.00
1930600
1930800
1931000
1931200
1931400
1931600
1931800
position (bp)

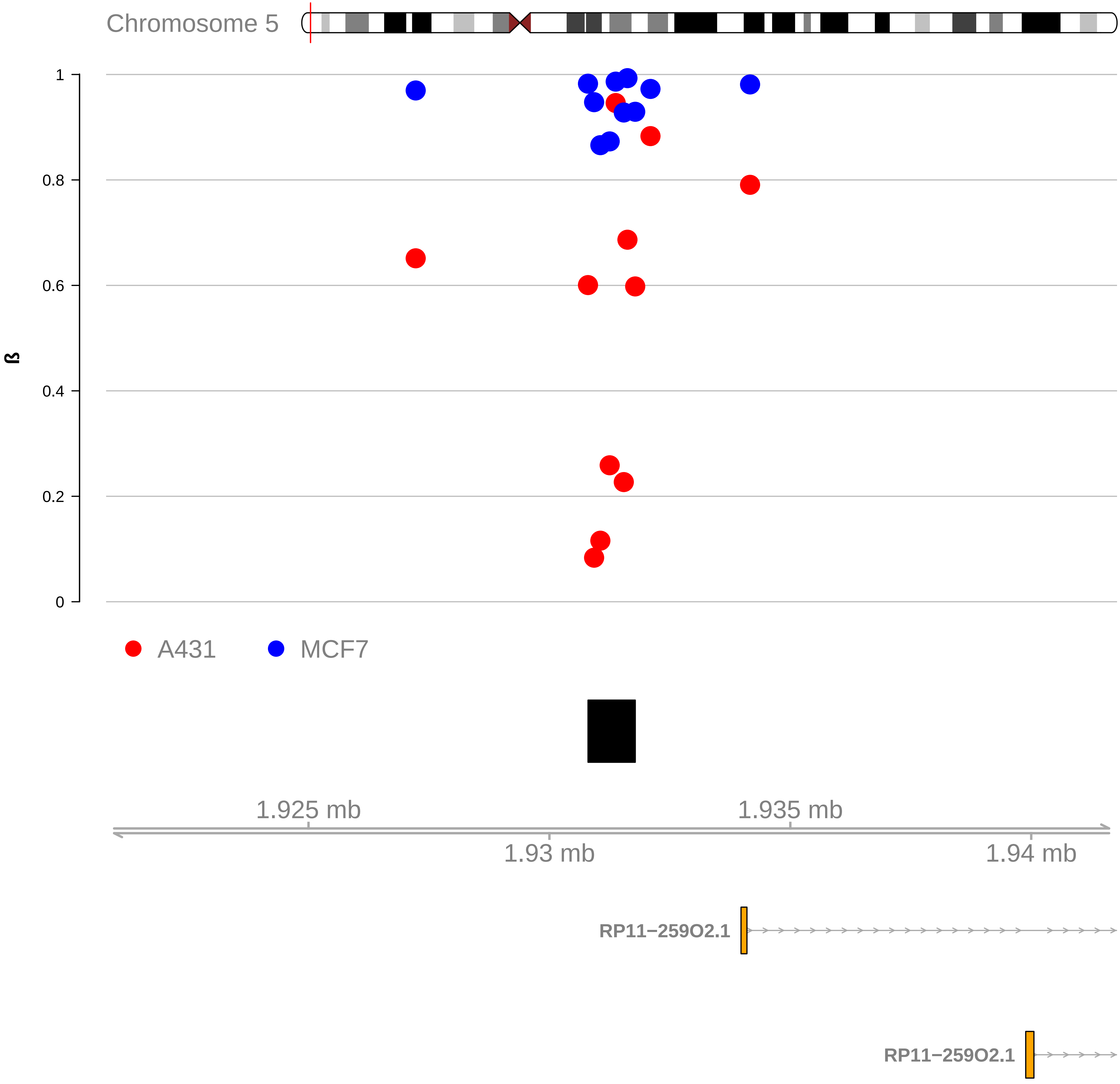

Chromosome 5
1
0.8
0.6
0.4
0.2
0
ß
A431
MCF7
1.925 mb
1.935 mb
1.93 mb
1.94 mb
RP11−259O2.1
RP11−259O2.1
